# Normal Reference Values of the Blackburne-Peel Ratio for Measuring Patellar Height in an Indian Population

**DOI:** 10.7759/cureus.37376

**Published:** 2023-04-10

**Authors:** Sejal Sanjeev Joshi, Avinash Dhok, Dipali Kadam, Kajal Mitra, Prashant Onkar

**Affiliations:** 1 Radiodiagnosis, NKP Salve Institute of Medical Sciences and Research Centre, Nagpur, IND; 2 Radiology, NKP Salve Institute of Medical Sciences and Research Centre, Nagpur, IND; 3 Radiodiagnosis and Imaging, NKP Salve Institute of Medical Sciences and Research Centre, Nagpur, IND

**Keywords:** blackburne-peel ratio, patellar instability, patella baja, patella alta, patellar height

## Abstract

Background

Historically, the quadriceps tendon has the largest sesamoid bone of the body, which is known as the patella. Patellar height is one of the important parameters in assessing patellar stability. The patella height has been found to vary in several diseases. As a result, ratios based on a variety of patellar bone indices are used to determine the norms. This study aimed to determine the typical patella height ratio in Indians, who have different sitting and squatting positions as opposed to Caucasians, by applying the Blackburne-Peel ratio to assess patellar height which is an alternative to the conventional Insall-Salvati ratio.

Methodology

A total of 100 normal lateral knee radiographs from the Indian population were used in this retrospective study. The Blackburne-Peel method (A/B) was used to calculate the ratios. It was calculated as perpendicular length measured from the inferior articular point of the patella to the point perpendicular (A) to the tibial plateau to the length of the articular surface of the patella (B).

Results

Patella height ratio for men was 0.67 ± 0.01, whereas, for women, it was 0.67 ± 0.02. The ratio did not differ significantly (p > 0.05) from the Western population.

Conclusions

The normal range of the Blackburne-Peel ratio for the Indian population was established, which can be used as a baseline and can be helpful in establishing patellar height in the Indian population. Similar to previous studies, our study shows that patella height ratios are stable regardless of gender or race and can be used to enhance and restore knee kinematics and functions.

## Introduction

The quadriceps tendon has the largest sesamoid bone of the body called the patella. It easily rolls over the femur articular surface [1]. The patella plays an important role in the extension activity of the knee by giving leverage to the quadriceps tendon [2]. Evaluation of anatomical alignment of the knee and assessment of knee pain along the anterior aspect requires radiological imaging of the patella [3]. The lateral radiograph of the knee was used by Blumensaat in 1938 to suggest a method for assessing patella height [3]. At least one line extending forward from the intercondylar notch served as a good reference point for placing the patella in its natural position. The ratio of patella length to maximal femoral condyle width was around 0.057 [4]. This ratio was selected by Blumensaat as its standard line ratio. Patella tendon length to patella length ratios were described by Insall and Salvati in 1971. Patella and patellar tendon lengths were found to be equivalent and to vary by no more than 20% on average, according to their research [5,6]. This was adopted as the Insall-Salvati ratio, which gained the approval of several researchers [7,8].

According to the height-to-age ratio utilized in pathological cases, lower or greater ratios have been recorded depending on the disease state. Examples include patella baja and chondromalacia, where the ratio is much lower than usual, and patella alta and Osgood-Schlatter disease, where the ratio is significantly higher than normal [9]. Patella height ratios of more than 1.3 indicate that the patella rides high, which is associated with a higher risk of patellar dislocations. The patella was also shown to be higher in athletes with chronic tendinopathy [10,11]. According to Agletti et al., even in patients who do not participate in athletics, changes in patella height from the norm are regarded to be permissive to injury [11].

When Blackburne and Peel proposed their study in 1977, they advocated for a revised patella height ratio. It can be used where the patella tendon is non-measurable because of a weak or an absent tibial tubercle such as in Osgood-Schlatter disease. Radiographing the patellar non-articulating lower half and its articulated upper half required slightly flexing the knee to 30 degrees [12]. This is how it came to be found, and it is now known to be constant. According to Blackburn and Peel, the aforementioned applies universally and is reproducible [11]. The Blackburne-Peel method is more reliable because it depends on more constant and consistent bony landmarks [10].

Hence, this ratio was employed to circumvent the difficulties arising from using the Insall-Salvetti Ratio. In our study, a lateral radiograph was used with the knee stretched at least 30 degrees to ensure that the slack in the patellar tendon was picked up. The tibial plateau was then measured twice along a line that projected forward. Its articular surface was called B, and A was the patellar articular surface perpendicular height from the tibial plateau line. Using the A/B ratio, patellar height was measured in our study [13].

The Blackburne-Peel ratio (A/B) was assessed in healthy Indian people to conclude its application and the occurrence of patella alta and baja in Indian residents, where squatting, sitting cross-legged, and kneeling are common [14].

## Materials and methods

**Figure 1:**
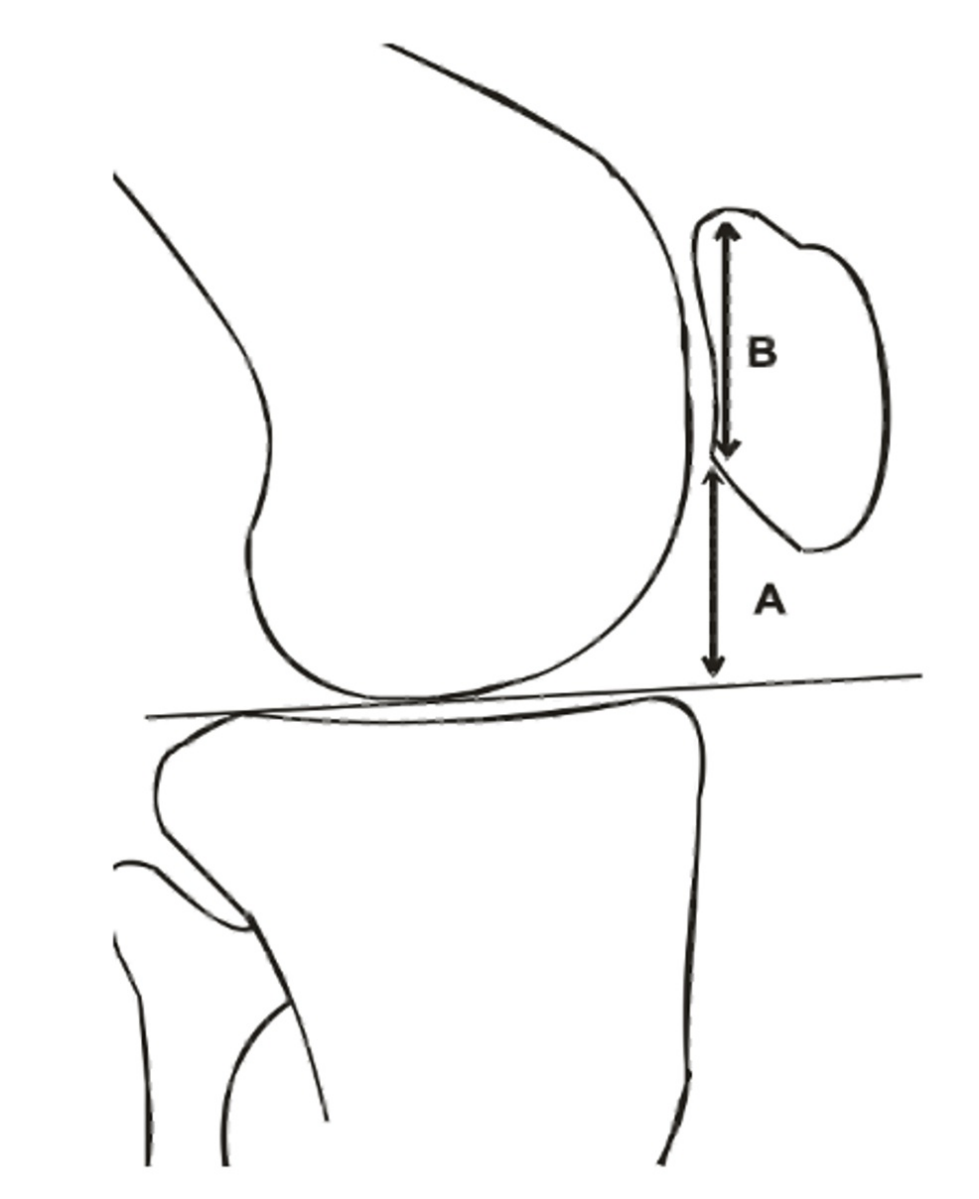
Diagrammatic representation demonstrating the length of the articular surface of the patella (B) and the tangential height of the inferior point of the articular surface of the patella from a horizontal line drawn along the tibial plateau (A). Blackburn-Peel ratio (A/B).

**Figure 2:**
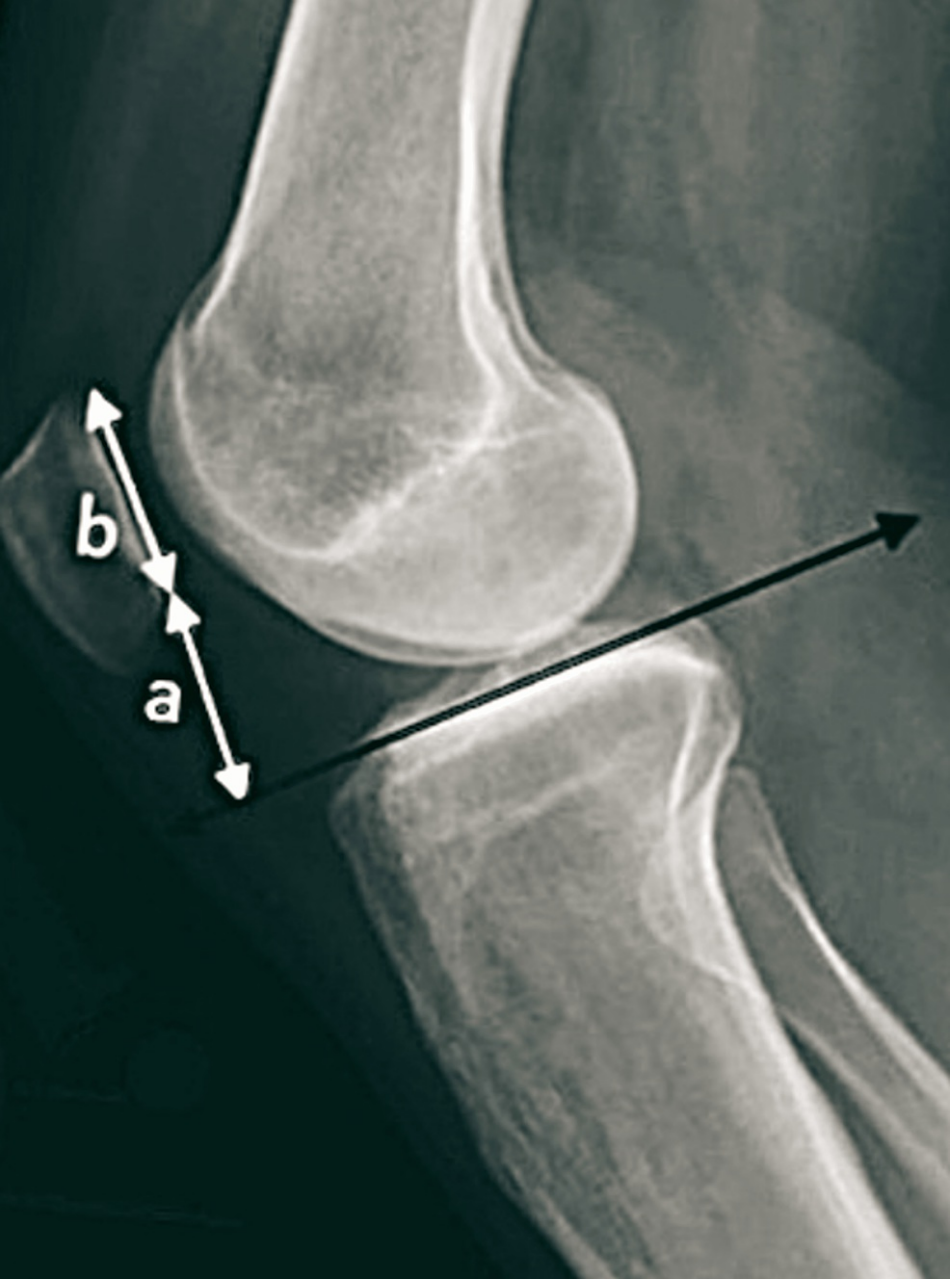
Radiographical demonstration of the Blackburn-Peel ratio (A/B) on a lateral knee radiograph.

A retrospective analysis of 100 normal lateral radiographs of the knee joints of adult men and women was conducted in the department of radiodiagnosis of a tertiary care hospital in central India. The lateral X-ray views of the knee were used to obtain all the data for this study. It is considered by equating the length of the two articular surfaces (A/B) to the angle created with the distal non-articular section of the patella and a streak tangent to the tibial plateau (Figures 1, 2). The gender and age of each radiograph were noted. The data were processed using 2019 Microsoft Excel. P-values <0.05 were used as the significance level.

## Results

This study included 51 male knee radiographs and 49 female knee radiographs over the age of 20. Table 1 and Table 2 include the demographic information of the participants. The mean A/B ratio for males and females is shown in Table 3. Our study findings failed to reach the level of significance (p > 0.05).

**Table 1:** Sex distribution of the participants.

Gender	N	%
Male	51	51
Female	49	49

**Table 2:** The Blackburne-Peel ratio (A/B) according to age and gender.

Age group (years)	Males	Females
20–24	0.65	0.65
25–29	0.65	0.65
30–34	0.67	0.66
35–39	0.68	0.68
40–44	0.68	0.69
45–49	0.67	0.68
50–54	0.69	0.69
55–59	0.68	0.68
60–64	0.68	0.68

**Table 3:** The mean A/B ratio among males and females.

Parameter	Male	Female	Blackburn and Peel (1977)	P-value
A/B ratio	0.67 ± 0.01	0.67 ± 0.02	0.80 ± 0.14	0.08

## Discussion

Patellar height is an important parameter in assessing patellar stability. Patellar height tends to be affected by various disease conditions [15]. Researchers have been trying to develop a simple, accurate, useful, and reproducible index to assess patellar height for decades [7].

In the 1930s, Blumensaat established a technique for determining patella height using lateral radiographs of the knee. The patella should normally be placed on or just above a line that extends forward from the intercondylar notch [3]. The patella length to maximum femoral condyle width ratio was calculated to be 0.95 ± 0.07. Several researchers also discovered that both factors were statistically very similar in terms of dimension. Blumensaat adopted this as its line ratio [6].

Insall-Salvati reported a ratio in 1971 that measured the length of the patella to the length of the patellar tendon on a lateral radiograph where the patella was under tension. The level of the patella appears to be correlated to its distance from femoral condyles in various pathological conditions, particularly patellar dislocation and chondromalacia. According to a study by Lancourt and Christi in 1975, using the Insall-Salvati ratio, the authors could associate conditions such as dislocation and chondromalacia with patellar height ratios. These conditions had a significantly low ratio of 0.8 whereas conditions such as Osgood-Schlatter disease had a high ratio of 1.2. Thus began the widespread use of the Insall-Salvati ratio [8].

The Insall-Salvati ratio may be popular because of its standard value of 1, which is easy to remember, or it may be popular because it has been used longer than other ratios [9]. However, it is not greatly reproducible, as reported by Berg et al. [14].

Indian population tends to have a habit of sitting cross-legged and squatting in their daily routines. The Insall- Salvati ratio, which is most popularly used in the calculation of patellar height, is often restricted to the Western population, which tends to have different sitting habits than Asians and may not be generalizable to others, as proved by studies done in China by Leung et al. and in India by Upadhyay et al. [16,17]. Hence, using the patellar height index values that have been derived from the Western population may not turn out to be useful in the Indian population [17].

The Blackburne-Peel ratio compares the distance of the articular cartilage surface of the patella to a line drawn from the tibial plateau to the inferior pole of the patella. While comparing the Blackburne-Peel ratio to the Insall-Salvati ratio, the former exhibits more interobserver reliability and can be used when the tibial tuberosity is abnormal or absent, as it eliminates the need to rely on this landmark [6].

Patella height was evaluated in our study using the Blackburn-Peel ratio that was derived from measurements made using lateral radiographs of the knees with the knee flexed to 30 degrees. The patella based on its height was described as patella baja if the height was smaller than normal, patella norma if the height was normal, and patella alta if the height was greater than normal [6]. The normal was 0.67 ± 0.01 in males and 0.67 ± 0.02 in females; the western standards are 0.80 ± 0.14.

The Blackburne-Peel method exchanges the reference point from the tibial tubercle to the tibial plateau. It was found that this was the most accurate and reproducible in comparison to the Insall-Salvati ratio. This ratio has been found not to be affected by the change in knee flexion from 300 to 500 [6].

To our surprise, we were able to obtain measurements in Indian participants that were quite close to those previously established for other ethnicities. The Blackburn-Peel ratio (A/B) was 0.67 ± 0.01 for men and 0.67 ± 0.02 for women, on average, according to the research. The average patellar height ratio did not fluctuate significantly between the two groups. This suggests that Indian patellar ratios are not sexually dimorphic. However, according to Leung et al., patella alta was more common in the South Asian population than in Caucasians in both control and the diseased, i.e., who did not have the symptoms related to the patella alta; hence, it was devised that the ratio for patella alta to be more than 3.4. This was not found in our study as our ratios were independent of race [16].

The ratios in Indians have been reevaluated in this study, and we believe that they are rather straightforward, helpful, and repeatable, regardless of ethnic variations. The restoration of patellar height is critical for proper knee function and kinematics, thus knowing the typical height of the patella is helpful during surgery [18]. According to Seil et al. and Berg et al., the patellar height index was most reliably replicated by the Blackburne-Peel ratio [13-19]. However, of note, only 22 knees and 15 people were included in the sample sizes for these two studies. In addition, no information was provided on the observers or their experiences in either study. Even though Lee et al. reported high dependability for the Blackburne-Peel and Insall-Salvati ratios, they did not disclose the characteristics of their two observers [20]. The quadricipital groove, formed by the ligamentum patellae pressure on the top end of the tibia, makes it difficult to project a line forward from the tibial plateau in squatters, as formerly addressed by Upadhyay et al. and Kate et al. [14-17]. It is possible that this influenced our findings about the Indian squatter community’s lower Insall-Salvati index dependability.

## Conclusions

In our study, there was no statistically significant variation in the typical patella height among the Indian and Western populations. The average values of the Blackburne-Peel ratio are almost the same among various races. Hence, the indices used in Caucasians to restore knee kinematics can be used in the Indian population.

## References

[1] Arthur FD, Keith LM (1999). Clinically Oriented Anatomy.

[2] Sutton D (1993). A Textbook of Radiology and Imaging. 5th edition, Churchill Livingston.

[3] Blumensaat C (1938). Die lageabweichungen und verrenkungen der kneischeibe. Ergebneisse Chir Orthop.

[4] Keats TE, Teeslink R, Diamond AE, Williams JH (1966). Normal axial relationships of the major joints. Radiology.

[5] Insall J, Salvati E (1971). Patella position in the normal knee joint. Radiology.

[6] Ahmed AD (1992). Radiological assessment of the patella position in the normal knee joint of adult Nigerians. West Afr J Med.

[7] Miller TT, Staron RB, Feldman F (1996). Patellar height on sagittal MR imaging of the knee. AJR Am J Roentgenol.

[8] Blackburne JS, Peel TE (1977). A new method of measuring patellar height. J Bone Joint Surg Br.

[9] Diederichs G, Issever AS, Scheffler S (2010). MR imaging of patellar instability: injury patterns and assessment of risk factors. Radiographics.

[10] Garms E, Teiveira de Carvalho R, Ramon LA, Matsuda MM, Cohen M (2011). Evaluation of the patellar height in athletes diagnosed with chronic tendripathy of the knee extensor mechanism. Acta Orthop Bras.

[11] Aglietti P, Insall JN, Cerulli G (1983). Patellar pain and incongruence. I: measurements of incongruence. Clin Orthop Relat Res.

[12] Levine AM, Drennan JC (1982). Physiological bowing and tibia vara. The metaphyseal-diaphyseal angle in the measurement of bowleg deformities. J Bone Joint Surg Am.

[13] Berg EE, Mason SL, Lucas MJ (1996). Patellar height ratios. A comparison of four measurement methods. Am J Sports Med.

[14] Kate BR, Robert SL (1965). Some observations on the upper end of the tibia in squatters. J Anat.

[15] Choudhary P, Bahre S (2017). Influence of prosthetic joint line position on outcome after total knee replacement. Int J Res Orthop.

[16] Leung YF, Wai YL, Leung YC (1996). Patella alta in southern China. A new method of measurement. Int Orthop.

[17] Choon DS (2013). Commentary: Position of the patella in adults in central India: evaluation of the Insall-Salvati ratio. J Orthop Surg (Hong Kong).

[18] Khakharia S, Scuderi GR (2011). Restoration of the distal femur impacts patellar height in revision TKA. Clin Orthop Relat Res.

[19] Seil R, Müller B, Georg T, Kohn D, Rupp S (2000). Reliability and interobserver variability in radiological patellar height ratios. Knee Surg Sports Traumatol Arthrosc.

[20] Lee PP, Chalian M, Carrino JA, Eng J, Chhabra A (2012). Multimodality correlations of patellar height measurement on X-ray, CT, and MRI. Skeletal Radiol.

